# Achieving optimal balance: tuning electrical and optical characteristics of carbon electrodes for emerging photovoltaics

**DOI:** 10.1039/d4ra01797h

**Published:** 2024-05-14

**Authors:** Amir Shehzad Gul, Muhammad Noman, Qandeel Rehman, Aimal Daud Khan, Muhammad Saad Rehan, Shayan Tariq Jan, Adnan Daud Khan

**Affiliations:** a U.S.-Pakistan Center for Advanced Studies in Energy, University of Engineering & Technology Peshawar Pakistan muhammad.noman@uetpeshawar.edu.pk

## Abstract

Transparent and conductive electrodes (TCEs) are essential for various optoelectronic and photovoltaic applications, but they often require expensive and complex fabrication methods. In this paper, a unique low-cost, eco-friendly, and scalable method of fabricating TCEs using spray-coated carbon ink is investigated. Firstly the carbon particles used for this process underwent a size reduction from 20 microns to 0.96 microns *via* ball milling. Then ink was prepared by mixing graphite powder (for conductivity), ethyl cellulose (for viscosity), and toluene (for solubility) with different weight-per-volume ratios (w/v) of 5%, 10%, and 15%. The TCEs were fabricated by spray coating the ink onto glass substrates using an airbrush. The sheet resistance (Ω sq^−1^) and transparency (%) of the TCEs were measured by a digital multimeter (DMM) probe method and a UV-vis spectrophotometer, respectively. The sheet resistance of the TCEs decreased linearly from 60 to 20 Ω sq^−1^, while the transparency decreased exponentially from 37.18% to 18.88% as the ink concentration increased from 5% to 15% w/v. This paper also reports the reflectance and absorbance values for each ink concentration. The results demonstrate that spray-coated carbon ink TCEs achieve sheet resistance and transparency values of 20 Ω sq^−1^ and 18.88%, respectively, with low-cost and eco-friendly materials and methods, which are desirable for optoelectronic and photovoltaic applications. These TCEs can play an important role as electrodes in semi-transparent perovskite cells enhancing their stability and overall efficiency.

## Abstract 2

This study pioneers a cost-effective, eco-friendly, scalable spray-coating technique for producing transparent & conductive electrodes with impressive electrical & optical performance, poised to boost the efficiency & stability of next-generation PV.

## Graphical abstract

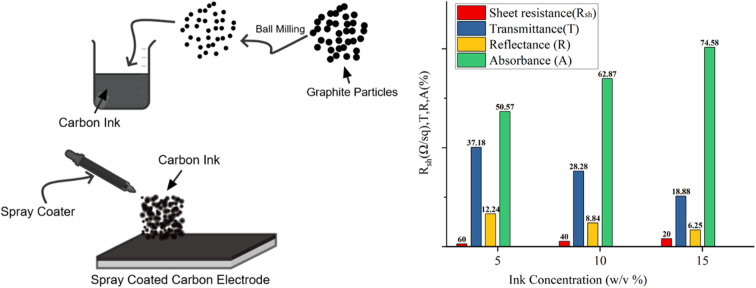

## 1. Introduction

Transparent conducting electrodes have been an area of interest for researchers and academics for a long time because of their numerous applications in technologies such as solar cells, photovoltaic cells, light-emitting diodes, touch-sensitive screens, flat-panel displays^1–8^ and perovskite solar cells.^9–17^ Although indium tin oxide (ITO) dominates the market for transparent conductive electrodes in the optoelectronic industry due to its high transparency (averaging 85% from 400 to 700 nm) and conductivity (with resistance as low as 10 per square) metal oxide,^18^ it has some restrictions as well. The restrictions include a requirement for work function alignment, high costs brought about by the scarcity of indium, demanding processing conditions involving high temperatures and vacuum environments, weak interfacial interaction because of its hard surface, and its fundamentally brittle and solid nature, which are major obstacles, particularly for flexible devices.^7,19–23^

Table 1 outlines the essential characteristics of several transparent conductive materials, contrasting them according to work function, cost, optical transmittance, and sheet resistance.

**Table 1:** Optical and electrical properties of various transparent conductive materials

Material	Sheet resistance (Ω sq^−1^)	Optical transmittance (%)	Work function (eV)	Cost ($ per m^2^)	References
ITO	10–100	80–90	4.5–5.0	100–200	21
Graphene	30–1000	85–97	4.5–5.0	10–100	24
AgNWs	10–100	80–95	4.2–4.6	10–50	25
CNTs	100–1000	80–95	4.5–5.0	1–10	26
Polymers	100–10000	70–90	3.0–5.5	<1	27

Researchers have studied a variety of materials in great detail to overcome these limitations and considered alternatives of ITO electrodes such as graphene,^28–30^ metal nanowires,^22,31–33^ carbon nanotubes,^26,34^ and conducting polymers.^35,36^ Carbon, in its various forms, serves an essential part as a material for electrodes owing to its distinctive physical, chemical, and electrical properties. There are many ways that carbon can appear, including carbon black, graphite, carbon nanotubes (CNTs), and graphene, all of which are attracting growing attention.^37^ Reduced graphite, exfoliated graphite, CNTs, and graphene are among the carbon forms that have shown excellent optoelectronic properties, such as high optical transparency, low sheet resistance, and high mobility. These carbon-based materials are also mechanically adaptable, environmentally safe, and reasonably priced, which makes them excellent alternatives for transparent electrodes.^38–40^ Therefore, research has concentrated on fabrication techniques, modification approaches, and patterning techniques to fully use the possibility of transparent electrodes based on CNTs and graphene for diverse optoelectronic devices.^12,13,41–43^

In this study, we examine how the weight-to-volume ratios of the carbon ink affect the electrical sheet resistance and optical transparency of spray-coated carbon electrodes. Our study analyzes how differences in ink composition affect the electrical and optical characteristics of carbon-based electrodes. Graphite is a relatively inexpensive and abundant material and is the main carbon source used in this study; it serves as the foundational element in the systematic development to create the appropriate electrodes. Reducing the size of graphite particles is the first stage in this procedure because it has a significant impact on how well electrodes work. We use planetary ball milling to convert bigger graphite particles into smaller, more uniform ones to achieve this size reduction. We then use the crushed graphite particles to make various weight-to-volume (w/v%) ratios of carbon ink formulations. Then, using a spray-coating procedure, the resulting carbon ink is applied to glass substrates. We anneal the electrodes on a hotplate to improve the adhesion and stability of the carbon ink layer. After fabrication, the characterization of the electrodes is carried out by evaluating their transparency with UV-vis spectroscopy and figuring out their electrical conductivity with sheet resistance testing. Furthermore, the X-ray diffraction (XRD) analysis is carried out to examine the structural characteristics of the carbon electrodes that were spray-coated.

This study broadens our insight into affordable and eco-friendly transparent electrodes, with a particular emphasis on the effects of differences in the ink composition. The purpose of this study is to show that spray-coated carbon-based electrodes can be a viable, affordable, and environmentally friendly option for high-performance, semi-transparent perovskite solar cells. We want to establish a desired balance between electrical conductivity and optical transparency by modifying the composition of the ink. This will allow for effective charge collecting while preserving a large amount of light transmittance for aesthetics and architectural integration.

## 2. Materials & methods: unique approach to carbon electrode fabrication

### 2.1. Materials

The following materials were used for the experiment: graphite powder (Sigma-Aldrich, USA; 99.9% purity; 20 μm particle size) served as the principal carbon source for electrode fabrication. Ethyl cellulose (EC) (Deajung, South Korea; 48% ethoxyl content) was used as the binder in the formulation of the carbon ink, which improved its adherence and stability. Toluene (RCI Labscan, Thailand; AR grade; 99.8% purity) served as the solvent for the preparation of the carbon ink, providing the right viscosity for consistent distribution. Glass slides (Sigma-Aldrich, USA; 3 cm × 3 cm) were chosen as the substrate material for electrode fabrication. Isopropanol or ethanol (Sigma-Aldrich, USA) was utilized to dilute the ink.

### 2.2. Methods

The four steps of the procedures employed in this study were as follows.

#### (A) Ball milling for particle size reduction

**Fig. 1:**
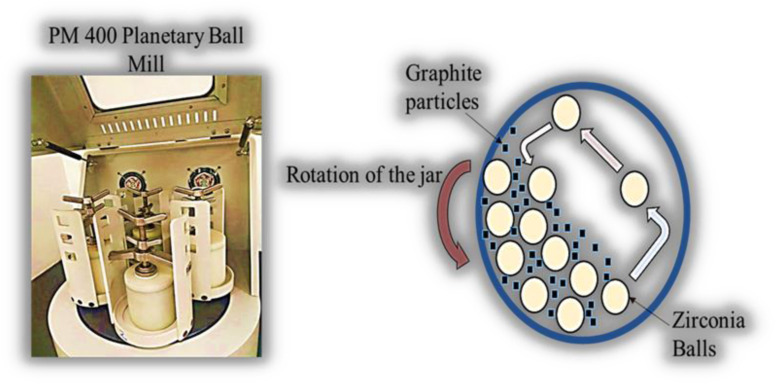
PM 400 planetary ball mill.

The first stage of our research concentrated on lowering the particle size of the graphite powder, which is an important component impacting electrode transparency. We used a planetary ball mill (Retsch PM 400) to achieve this decrease. Larger graphite particles were mechanically converted into smaller, more uniform substitutes during ball milling. An illustration of the PM 400 planetary ball mill, which was employed in the study to reduce the particle size is depicted in Fig. 1.

A ball mill is used in the procedure to produce the required level of fineness for the finished product. A ball mill is a cylindrical chamber that rotates around its axis while containing a grinding medium, such as balls.^44^ First, around 20 g of graphite powder (Sigma-Aldrich, 99.9% purity, a 20 micron particle size) was taken into the jar of the ball mill. Then, two times the weight of the graphite powder, zirconia balls (Sigma-Aldrich, 95% purity, 10 mm diameter) were placed in the jar. The graphite powder-to-balls ratio was maintained at 1 : 2. The ball milling was then performed at about 350 rpm for 6 hours during the first day using a planetary ball mill (Retsch PM 400). The machine was given rest for about 10 minutes after each hour to avoid heating issues; the process remained continuous for at least 4 days with 6 hours of milling each day. After each milling session, we monitored the particle size using a Zeta Sizer. Following the initial milling, we repeated the ball milling process three times with the same parameters. The ball-milled graphite powder was then collected in a closed container and stored for further application. The next step in the process is the preparation of the carbon ink from the powder.

#### (B) Carbon ink formulation

**Fig. 2:**
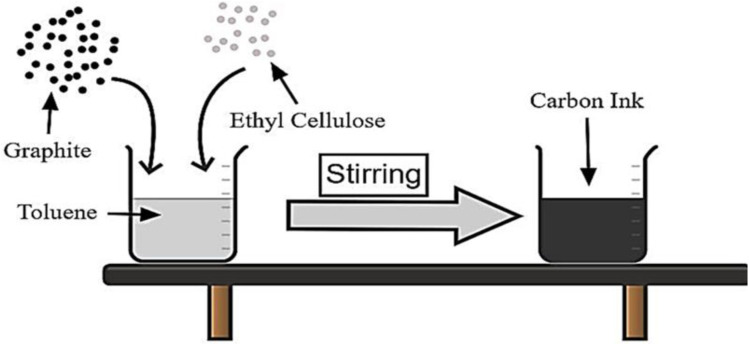
Carbon ink formulation process.

The carbon ink formulation, a critical aspect of our research, required careful blending of minimized graphite powder with ethyl cellulose binder and toluene solvent. This procedure guaranteed that a consistent ink composition with defined qualities was created, which contributed to uniform ink coating in electrode fabrication. An overview of the formulation process for carbon ink, demonstrating the blending of toluene, ethyl cellulose, and graphite at various weight-to-volume ratios is shown in Fig. 2.

The creation of consistent, quality ink for later electrode fabrication required a specific set of actions during the preparation of carbon ink. The chemical composition was made by combining ethyl cellulose, toluene, and powdered graphite that had been ball-milled at various ratios. The three different weight/volume (w/v) proportions of carbon ink comprised 5%, 10%, and 15%. The various weight-to-volume ratios of carbon ink formulations are shown in Table 2 along with the appropriate amounts of toluene, ethyl cellulose, and graphite used for each.

**Table 2:** Details of carbon ink formulations

Ink concentration (w/v%)	Graphite (g)	Ethyl cellulose (g)	Toluene (ml)
5	5	2.5	100
10	10	5	100
15	15	7.5	100

For consistency's sake, the weight ratio of ethyl cellulose (EC) to graphite was kept constant at 1 : 2 for all ink concentrations (this standard ratio made sure that the attributes of the producing carbon ink remained constant). The amount of ethyl cellulose (EC) can be reduced slightly if the ink is too thick for deposition. For the initial concentration of 5% (w/v), we dissolved 5 g of ball-milled graphite particles in 100 ml of toluene. The additional ink concentrations were made using the same methodology, preserving the constant graphite-to-toluene ratio. The ink components were thoroughly mixed for approximately two hours by stirring. The properly made and uniform carbon ink was then gently poured into a glass container and capped.

#### (C) Electrode fabrication

**Fig. 3:**
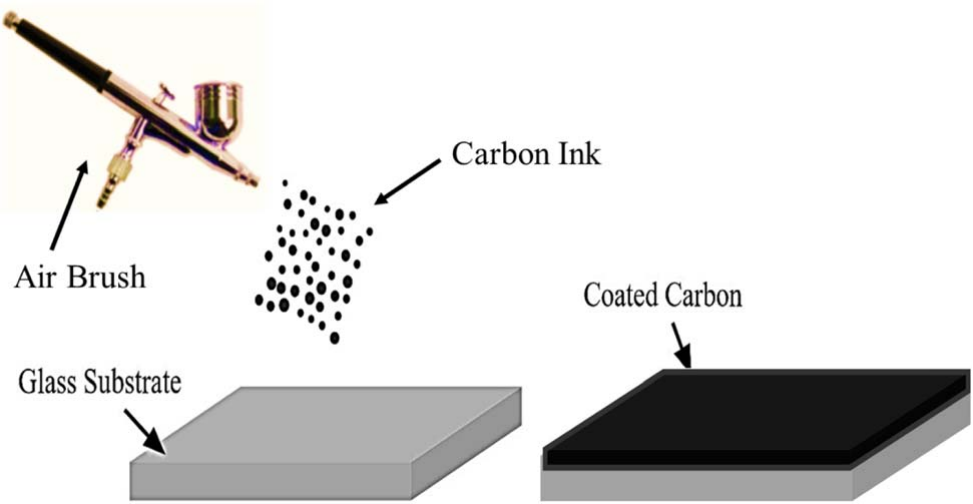
Crafting electrodes through spray coating.

We used a precision spray-coating approach to make the carbon electrodes using airbrush. The produced carbon ink was sprayed uniformly on glass substrates. This stage, which is essential for ensuring even ink distribution, lays the basis for further characterization. An illustration of the carbon electrode creation process on glass substrates using spray coating is shown in Fig. 3.

Employing a precise spray-coating process, prepared carbon ink is applied to glass substrates to create carbon electrodes. This phase is essential for guaranteeing the carbon layer's homogeneity and adherence to the substrate. Glass slides with 3 cm × 3 cm surface areas were chosen as the substrate. To get rid of any impurities or remains that would interfere with the carbon ink's ability to adhere, these glass slides were extensively washed using deionized water and acetone. The carbon ink was applied to the ready glass substrates using an airbrush, a precision spray-coating device. With the airbrush, the deposition process could be precisely controlled, resulting in uniform coverage. The spray-coating device was loaded with carbon ink, which had been carefully controlled for composition and concentration. The ink was thoroughly mixed and checked for any air bubbles that would cause uneven application. The glass substrate with the applied ink underwent annealing after the carbon ink was deposited on it. To improve the adherence and durability of the carbon ink layer, the substrates were heated at 100 °C on a hotplate for 30 minutes. To avoid contamination or damage, the finished carbon electrodes were kept in a controlled atmosphere.

#### (D) Characterization of electrodes

Since the graphite particles underwent ball milling, the size of reduced particles was examined by Zeta Sizer. X-ray diffraction (XRD) analysis was used to examine the structural characteristics of the carbon electrodes that were spray-coated. To determine the crystalline structure of the sample, which was generated with an optimized 10% w/v ink concentration and a particle size of 0.96 microns, XRD analysis was performed. UV-vis spectroscopy was used to evaluate the optical transparency of the carbon electrodes. A sheet resistance test was performed to assess the sheet resistance of the electrodes by employing a digital multimeter. The probe method was used to assess the sheet resistance of the carbon film electrode. This method involves applying a known current through two outer probes and measuring the voltage drop across two inner probes. The DMM then calculates the resistance using Ohm's law.

**Fig. 4:**
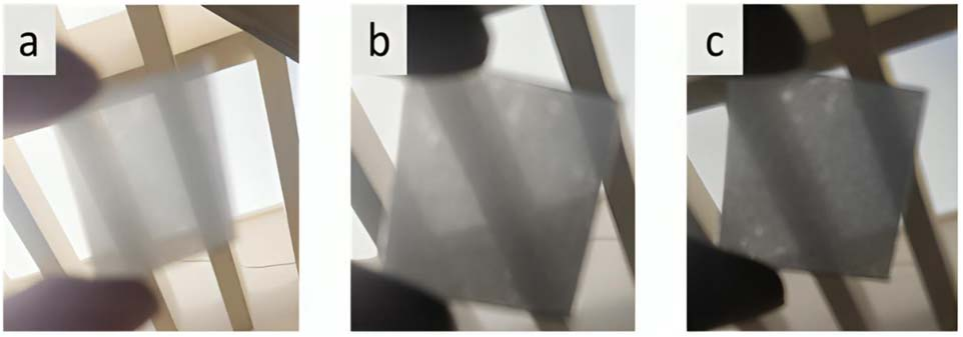
Carbon-coated glass slides at (a) 5%, (b) 10%, and (c) 15% concentrations.

The below illustration in Fig. 4(a)–(c) shows the carbon-coated glass slides with different concentrations of 5%, 10% and 15% respectively.

UV-vis spectroscopy was used to gauge how transparent the carbon electrodes were optically. This method offers important insights into the efficiency of light transmission *via* the electrodes, a vital aspect of their prospective usage in optoelectronic devices. To ascertain the electrical conductivity of the manufactured carbon electrodes, sheet resistance measurements were performed. This is an essential characteristic to assess how well they conduct electrical current. The measurements of sheet resistance shed light on the electrodes' ability to promote electron flow, which is crucial in many electrical and solar applications.

## 3. Results and discussion

This work explored the use of spray-coated carbon ink as a low-cost, sustainable, and scalable method for producing transparent, conductive electrodes. The study aimed to investigate the effects of decreasing graphite particle content in ink on the optical and electrical characteristics of carbon-based electrodes.

### 3.1. Particle size analysis

Before using the graphite particles to create carbon ink, they were ball-milled. The technique section provides a detailed description of the milling process. To look at the variations in particle size that happened during the ball milling process, Zeta Sizer tests were done. These results contribute to our knowledge of the process of size reduction, which is an important issue in our study.

**Fig. 5:**
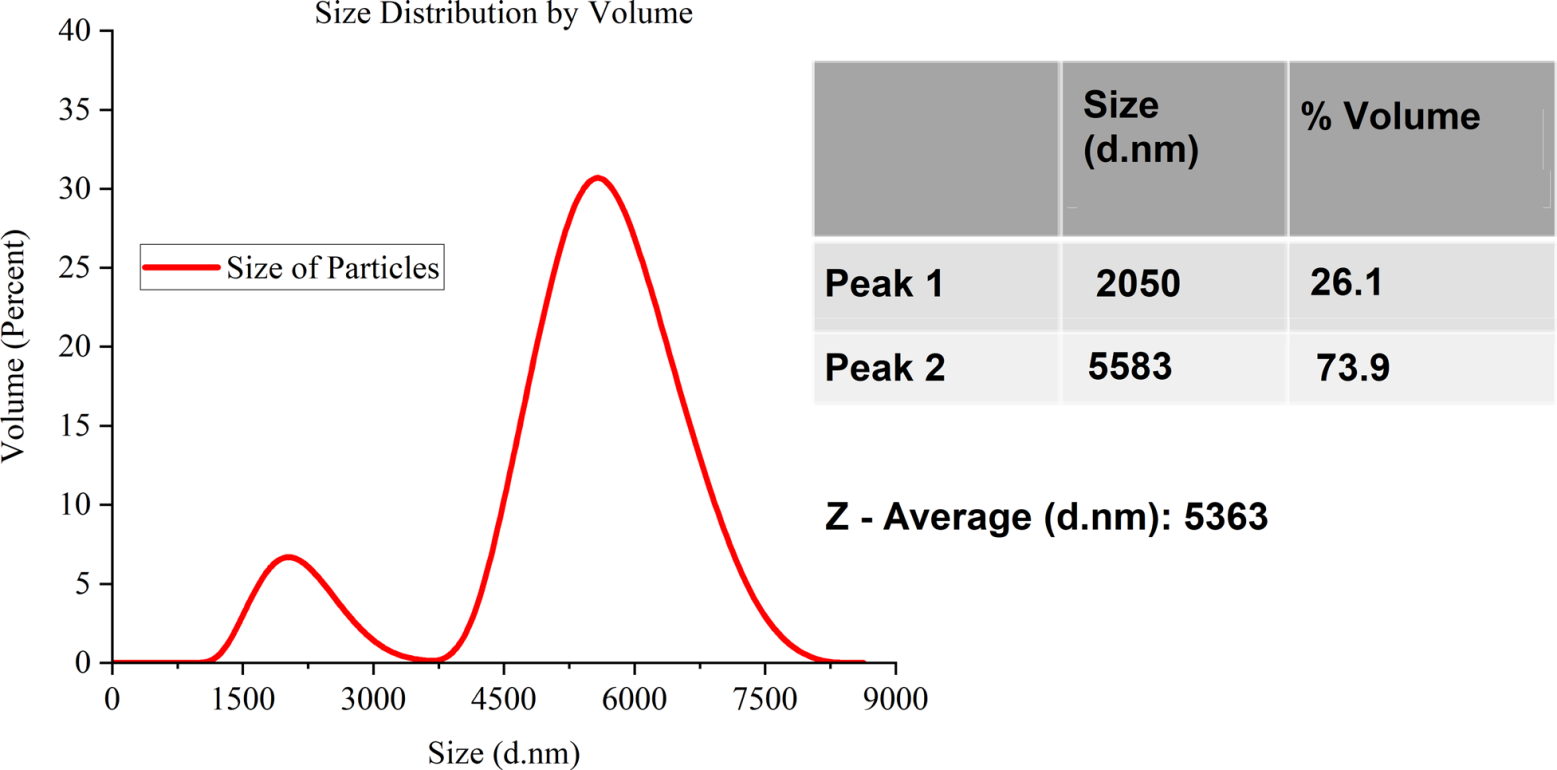
Carbon particle size distribution determined by Zeta Sizer.

**Fig. 6:**
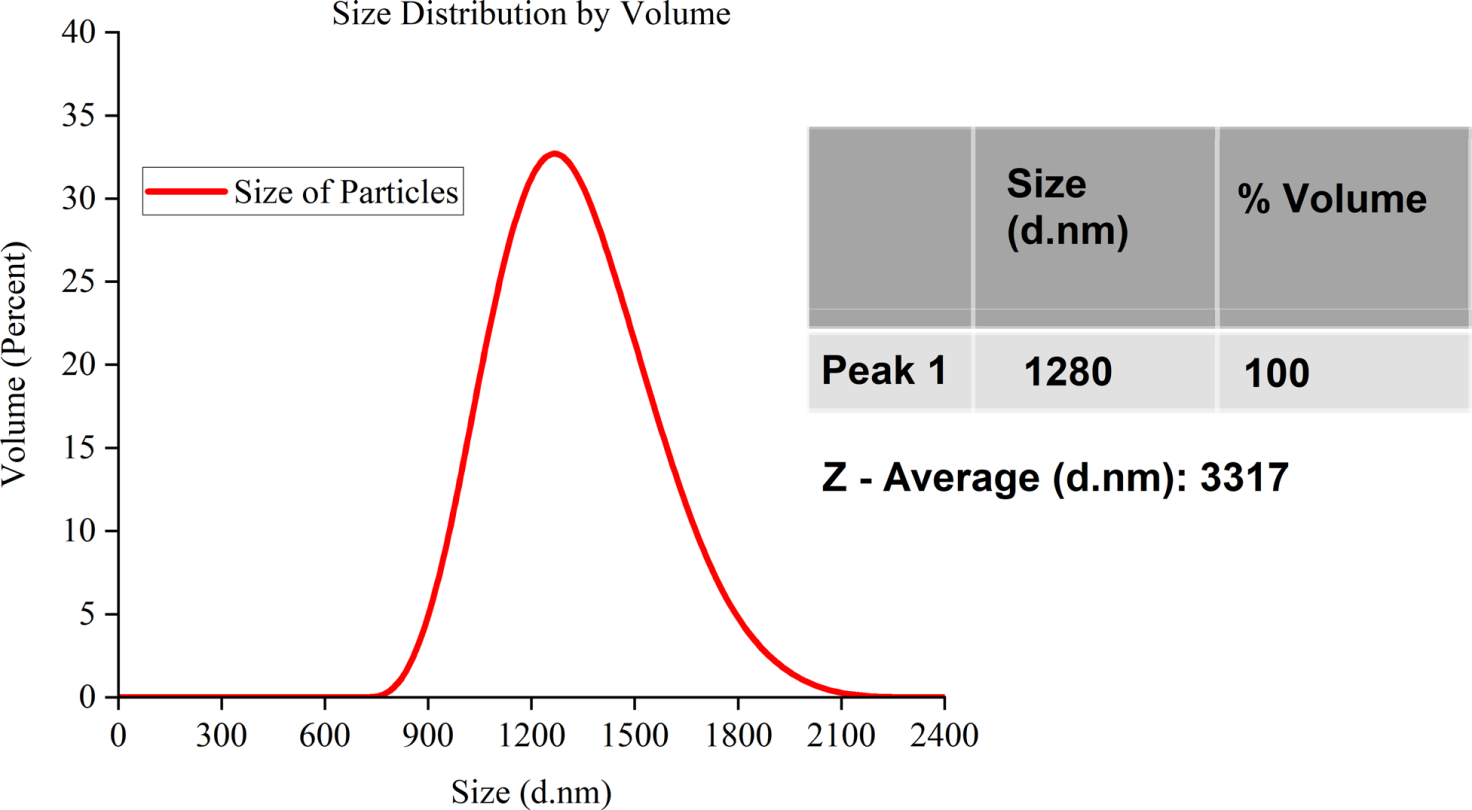
Carbon particle size distribution after a second milling cycle.

**Fig. 7:**
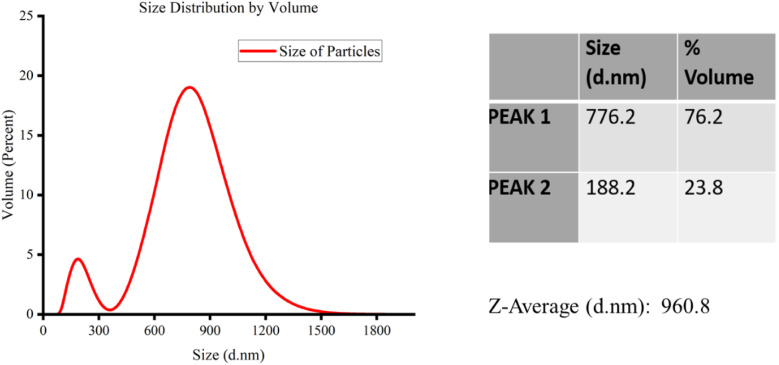
Carbon particle size distribution after a third round of milling.

The graphite particles were first ball-milled before they were used to make carbon ink. The milling process is described in detail in the methodology section. Fig. 5–7 shows the Zeta Sizer results after each session of ball milling.

According to the Zeta-Sizer data, the carbon particles in Fig. 5 have a bimodal size distribution, which suggests that there are two distinct groups of particles with varying sizes. The average size of the first peak, which accounts for 26.1% of the overall volume, is 2050 nm. The average size of the second peak, which accounts for 73.9% of the overall volume, is 5583 nm. The *Z*-average wavelength is 5363 nm. It is a weighted mean based on dispersed light intensity.

The findings in Fig. 6 were obtained after submitting the previously ball-milled particles to another round of milling. The results show how much the size of the carbon particles dropped following the second round of milling. The size distribution is now unimodal, which means that there is just one group of particles that are all the same size. The average particle size in this group is 1280 nm, indicating that the milling process has broken down the bigger particles into smaller ones. The volume proportion of this category is 100%, suggesting that the sample lacks any other size classes. The *Z*-average size is 3317 nm, and it is still based on scattered light intensity.

Fig. 7 shows the results of a third cycle of milling on the carbon particles. The results show that the carbon particle size was reduced even further during the third round of milling. The size distribution is bimodal once more, showing the presence of two separate particle groups of differing sizes. The average size of the first group's peak, which accounts for 76.2% of the total volume, is 776.2 nm. The average size of the second group's peak, which accounts for 23.8% of the total volume, is 188.2 nm. The weighted mean, or *Z*-average size, is determined by the intensity of dispersed light and is 960.8 nm.

Table 3 illustrates how the particle size gradually decreases during the milling cycles. The dynamic variations in particle size are shown by the bimodal distributions that shift from unimodal to bimodal after each milling cycle. The *Z*-average size consistently decreased across the cycles, highlighting the milling process's ability to precisely adjust particle sizes, a critical step in maximising the carbon particles' qualities for the intended uses. The results show that milling is highly crucial to obtaining the appropriate particle size distribution for particular industrial applications.

**Table 3:** A summary table of the carbon particle size distribution from the Zeta-Sizer data across the three milling cycles

Figure	Milling cycle	Distribution type	Peak 1 size (nm)	Peak 1 volume (%)	Peak 2 size (nm)	Peak 2 volume (%)	*Z*-Average size (nm)
Fig. 5	Initial	Bimodal	2050	26.1	5583	73.9	5363
Fig. 6	Second	Unimodal	1280	100	N/A	N/A	3317
Fig. 7	Third	Bimodal	776.2	76.2	188.2	23.8	960.8

### 3.2. X-ray diffraction (XRD) analysis

**Fig. 8:**
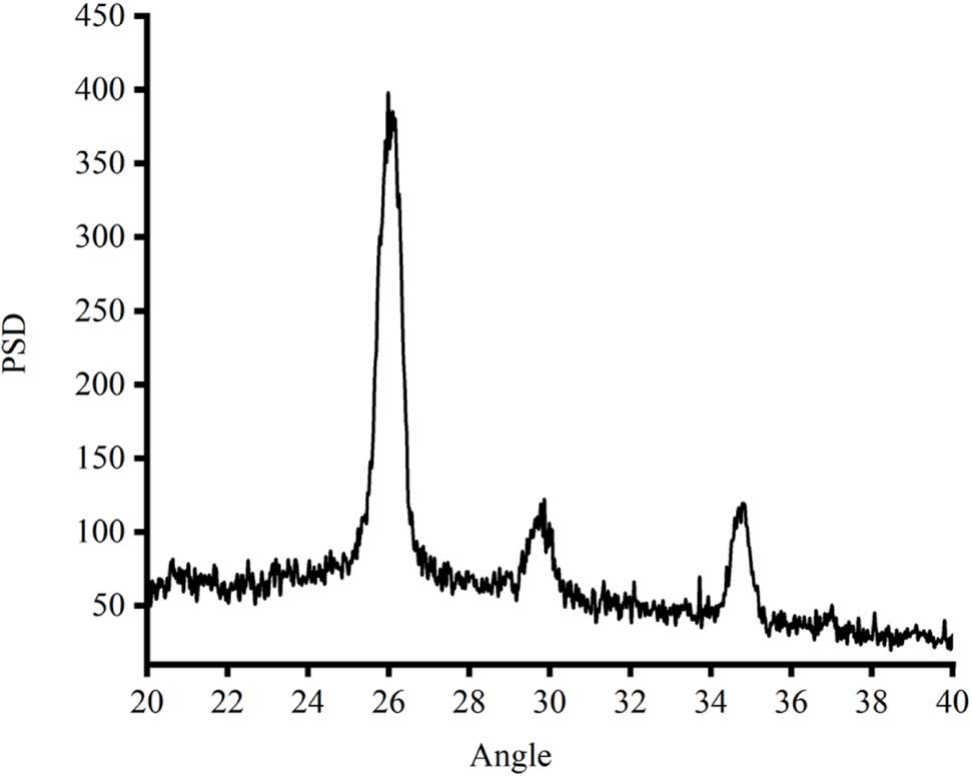
XRD pattern of the spray-coated carbon film electrode.

The XRD spectrum of the spray-coated carbon electrodes designed for perovskite solar cells, as presented in Fig. 8, exhibits pronounced peaks that signify a well-defined crystalline nature of the carbon film. The dominant peak at an angle of 26° signifies the presence of a graphitic-like structure within the electrode.^45^ In comparison to the graphitic peak, other observed peaks in the spectrum are notably lower in intensity, which might correspond to different forms of carbon or various crystalline orientations within the film.

Mirroring the graphitic peak's significance, the electrode's crystallinity, as indicated by the sharpness of this peak, suggests efficient electrical pathways that are conducive to electron transport. Crystalline structures with ordered arrangements of carbon atoms facilitate the movement of electrons through delocalized orbitals. This efficient electron transport translates to higher electrical conductivity within the electrode, which is crucial for effective charge collection in solar cells. This crystallinity could also result in favourable optical properties. The high degree of crystallinity suggested by the sharp peak implies a well-organized structure. Light encounters less disruption as it travels through this organized film compared to an amorphous structure with a random arrangement of atoms. This reduced light scattering leads to increased optical transparency, allowing more light to pass through the electrode and reach the active layer of the solar cell. Additionally, the low background noise and the clear baseline of the XRD pattern affirm the precision of these measurements, enhancing the reliability of the interpretation.

The XRD spectrum shows secondary peaks alongside the dominant graphitic peak at 26°. These secondary peaks are indicative of additional crystallographic planes and structural defects within the carbon material. The presence of minor crystalline phases besides graphite influences the overall electrical and optical properties of the electrodes. For instance, some non-graphitic phases have lower conductivity compared to graphite, affecting the bulk conductivity of the electrode. The presence of a broad amorphous phase peak suggests a less ordered structure, which also impacts conductivity and scatter light more, reducing transparency. Studies have shown that amorphous carbon structures tend to have higher resistivity and lower optical transparency compared to their crystalline counterparts.^24^ Crystallographic defects also act as scattering centres for electrons, potentially hindering their mobility and reducing conductivity. The impact of defects on electron transport has been well-documented in the literature, highlighting their role in modifying the electrical properties of carbon-based materials.^25^

### 3.3. UV-vis spectra analysis

**Fig. 9:**
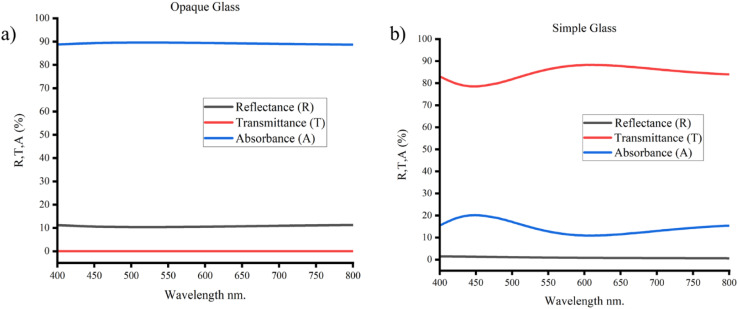
UV-vis spectra of the (a) opaque and (b) simple glass slide.

The UV-vis spectra of a simple, opaque glass slide are shown in Fig. 9. The plain glass slide in Fig. 9(b) has a high transmittance of more than 90% in the visible spectrum compared to the opaque glass in Fig. 9(a) which has a very low transmittance of almost equal to zero. The glass slides' reflectance and absorbance are also considerably different. The opaque glass slide has a high absorbance of more than 85% and a low reflectance of less than 10% compared to the simple glass slide which has an absorbance of less than 20% and reflectance of almost zero. These findings suggest that the performance of the electrodes can be impacted by the substrate material's optical characteristics.

**Fig. 10:**
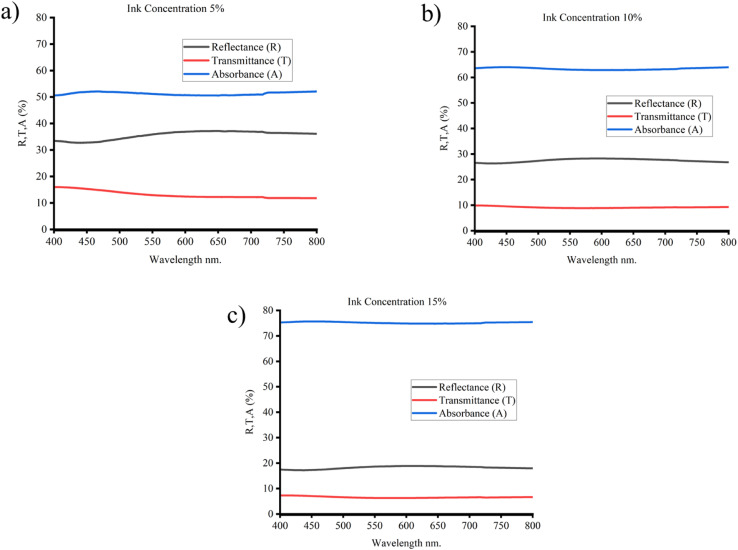
UV-vis spectra of carbon electrodes with (a) 5%, (b) 10% w/v and (c) 15% w/v ink concentration.

The UV-vis spectra of carbon electrodes with various ink concentrations are shown in Fig. 10. The graphs clearly show the trends of declining transmittance and rising absorbance for a given size as ink concentration rises. The findings show that the sheet resistance and transparency of spray-coated carbon ink electrodes are significantly influenced by ink concentration. The results indicate that both particle size and ink concentration have significant effects on the sheet resistance and transparency of spray-coated carbon ink electrodes.

The results also demonstrate that as particle size reduces from 20 microns to 0.96 microns, the optical transparency of the electrodes drastically rises. The reduction in light scattering caused by tiny particles, thereby improving the optical transmittance of the electrodes, is the cause of a rise in transparency as the particle size decreases.^46^ Similarly, as the ink concentration increases, the number of light-absorbing particles (carbon) per unit area in the electrode film also increases.^6^ This leads to stronger absorption of light across the entire UV-vis spectrum, resulting in a higher overall absorbance value in Fig. 10. The ink concentration also affects the charge carrier mobility within the electrodes. Higher ink concentrations typically result in thicker electrode layers with a higher volume fraction of carbon material. This denser structure leads to enhanced charge carrier mobility due to improved percolation pathways for electron transport. This denser structure can facilitate charge carrier mobility by providing more robust percolation pathways for electron transport, as has been demonstrated in previous studies. In summary, increasing ink concentration enhances light absorption while also potentially improving charge carrier mobility. These results are in line with earlier research that found comparable impacts of particle size on the sheet resistance and transparency of carbon-based electrodes.^40,47^

### 3.4. Sheet resistance analysis

The carbon electrodes' average sheet resistance ranged from 20 to 60 Ω sq^−1^, and their average transparency in the visible spectrum ranged from 18.88% to 37.18%. The sheet resistance and transparency results for each sample of carbon electrodes with various ink concentrations and particle sizes are summarized in Table 3.

**Fig. 11:**
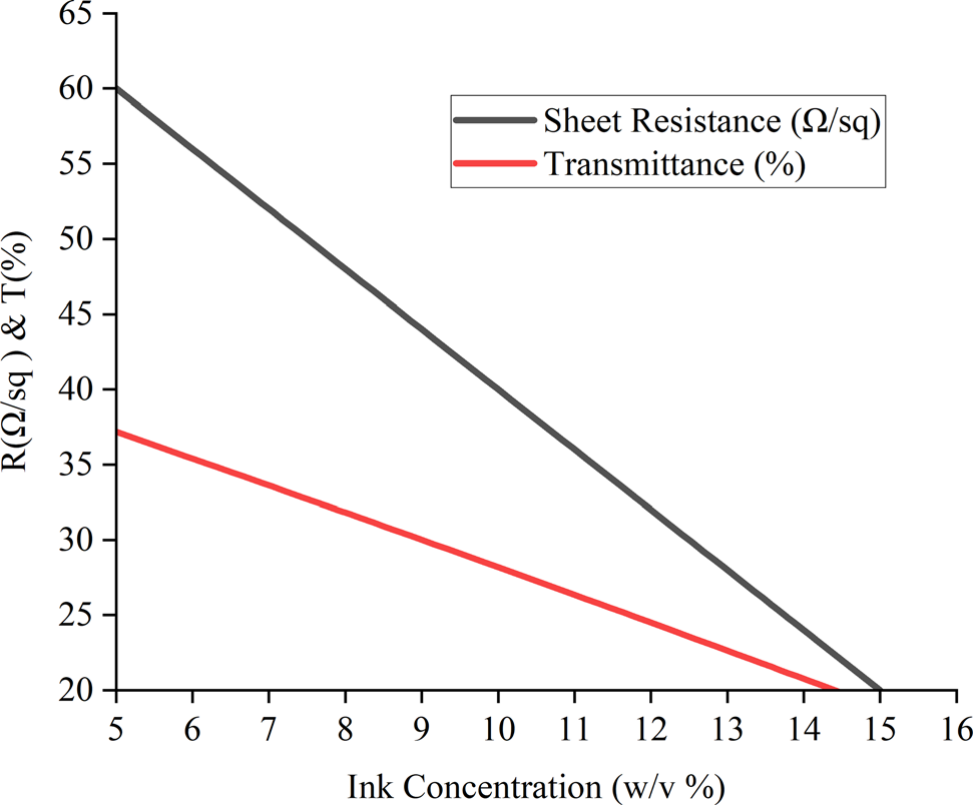
Graph of sheet resistance and transmittance against ink concentration.

The corresponding trend between sheet resistance and transparency against the ink concentration from the respective table is depicted in Fig. 11.

Table 4 also displays a distinct pattern that appears when the sheet resistance and optical characteristics of carbon electrodes with different ink concentrations are examined. The resistance of the electrodes drops dramatically when the ink concentration rises from 5% to 15% (w/v), with the lowest sheet resistance of 60–70 Ω sq^−1^ and the highest concentration of 20–30 Ω sq^−1^.

**Table 4:** Sheet resistance and optical properties of carbon electrodes with different ink concentrations

Ink concentration (% w/v)	Sheet resistance (Ω sq^−1^)	Transmittance (%)	Reflectance (%)	Absorbance (%)
5	60–70	37.18	12.24	50.57
10	40–50	28.28	8.84	62.87
15	20–30	18.88	6.25	74.58

This suggests that the connection between sheet resistance and ink concentration is inverse, meaning that larger ink concentrations improve the electrodes' electrical conductivity. At the same time, there is a comparable change in optical properties: transmittance goes from 37.18% to 18.88%, reflectance from 12.24% to 6.25%, and absorbance from 50.57% to 74.58%. By reflecting less light and absorbing more, these alterations suggest that electrodes with higher ink concentrations are less transparent. This might be especially helpful in situations when it's preferable to have low sheet resistance and strong light absorption, such as in sensors or solar systems.

Research has shown a direct correlation between decreased electrical resistance and smaller particle sizes. As the particle size drops from 20 microns to 0.96 microns, the electrodes exhibit a considerable reduction in resistance. The decrease in sheet resistance as the particle size decreases can be explained by the increase in contact area between the carbon particles, which enhances the electrodes' electrical conductivity.^48^

The sheet resistance of the carbon electrodes decreases as the ink concentration increases, regardless of the particle size. As ink concentration rises from 5% to 15% w/v, the sheet resistance noticeably reduces. The increase in the volume fraction and packing density of graphite particles is the main cause of this phenomenon. As ink concentration rises, the likelihood of continuous electric current paths increases, resulting in increased conductivity.^49^ Simultaneously, the transparency of carbon electrodes decreases with increasing ink concentrations, again independent of particle size. The research indicates a decline in electrode transparency as the concentration of ink moves from 5% to 15% w/v. This occurs as a result of the higher carbon content causing an increase in thickness and opacity.^50^Table 5 shows the sheet resistance and optical transmittance of the carbon electrodes with those of other commonly used transparent conductive materials.

**Table 5:** Sheet resistance and optical transmittance of transparent conductive materials

Material	Sheet resistance (Ω sq^−1^)	Optical transmittance (%)	References
ITO	10–100	80–90	21
Graphene	30–1000	85–97	24
AgNWs	10–100	80–95	25
CNTs	100–1000	80–95	26
Polymers	100–10000	70–90	27
**Spray-coated carbon ink electrodes (this work)**	**20–60**	**18–37**	(Current manuscript)

According to the study's findings, flexible electronics and solar systems that require low-cost, environmentally friendly, and scalable production techniques can use spray-coated carbon ink electrodes.^51^ Transparent and conductive electrodes can be produced in large quantities and at high throughput using the spray coating method on a variety of substrates. The carbon ink electrodes have several advantages over conventional materials like indium tin oxide (ITO) or silver nanowires (AgNWs), such as lower cost, higher availability, better stability, and greater flexibility. However, they also have some drawbacks, such as lower transparency when compared to ITO or AgNWs or higher resistance when compared to electrodes made of metal.^52,53^ These findings imply that spray-coated carbon ink electrodes, which provide a compromise between electrical conductivity and optical transparency, may find application in semi-transparent perovskite solar cells.^50^ Future research will include these electrodes in perovskite solar cells and assess how they affect the device's overall performance and power conversion efficiency.^54^

The findings of this study demonstrate that the concentration of ink and the size of the graphite powder particle affect the sheet resistance and transparency of spray-coated carbon ink electrodes. As the particle size is reduced, the resistance falls and the transparency rises. As the ink concentration rises, the sheet resistance and transparency likewise decline.

## 4. Conclusion

This work assessed the effects of ink concentration and particle size on the optical and electrical characteristics of carbon ink electrodes coated with a spray coating, with an emphasis on photovoltaic and optoelectronic uses. Our findings demonstrated that resistance decreases and transparency increases with decreasing particle size. Reactivity and transparency also decrease with increasing ink concentration. Regardless of the size of the particles, the resistance of the carbon electrodes diminishes as the ink concentration rises. Increasing the ink concentration from 5% to 15% w/v causes a discernible decrease in sheet resistance. This effect is primarily caused by a rise in the volume fraction and packing density of graphite particles. Particle size again does not affect the simultaneous decrease in transparency of the carbon electrode that occurs with increasing ink concentrations. According to the study, electrode transparency decreases when ink concentration rises from 5% to 15% w/v. This happens because of the increased thickness and opacity brought on by the higher carbon concentration. The average sheet resistance of the carbon electrodes varied between 20 and 60 Ω sq^−1^, and their average transparency in the visible spectrum varied between 18.88% and 37.18%. Our findings validated the percolation hypothesis and demonstrated the adaptability of this technique in terms of controlling electrode resistance and transparency. In particular, these adjustable features indicate a promising future for semi-transparent perovskite solar cells, where a careful balance between light absorption and electrical conductivity is needed.

## Data availability

The data is available from the corresponding author on reasonable request.

## Conflicts of interest

The authors have no competing interests to disclose.

## Supplementary Material
